# Dynamic Remodeling of the Host Cell Membrane by Virulent Mycobacterial Sulfoglycolipid-1

**DOI:** 10.1038/s41598-019-49343-2

**Published:** 2019-09-06

**Authors:** Manjari Mishra, Pranav Adhyapak, Ruchika Dadhich, Shobhna Kapoor

**Affiliations:** Department of Chemistry, Indian Institute of Technology Bombay, Powai, India

**Keywords:** Membrane biophysics, Membrane structure and assembly, Cellular imaging

## Abstract

Lipids dictate membrane properties to modulate lateral membrane organization, lipid/protein diffusion and lipid-protein interactions, thereby underpinning proper functioning of cells. *Mycobacterium tuberculosis* harnesses the power of its atypical cell wall lipids to impact immune surveillance machinery centered at the host cell membrane. However, the role of specific virulent lipids in altering host cellular functions by modulating membrane organization and the associated signaling response are still pertinent unresolved questions. Here, combining membrane biophysics and cell biology, we elucidate how virulent *Mtb* sulfoglycolipids hijack the host cell membrane, affecting its order, fluidity, and stiffness along with manipulating the linked cytoskeleton. The functional outcome of this perturbation was assayed by monitoring membrane-associated autophagy signaling. These actions form a part of the overall response to commandeer host membrane-associated immune processes during infection. The findings on the mechanism of action of *Mtb* lipids on host cell membrane structure and downstream signaling will deepen the collective understanding of their functional aspects in membrane-dictated bacterial survival, pathogenesis and drug resistance and reveal suitable membrane driven-therapeutic intervention points and diagnostic tools.

## Introduction

The emergence of antibiotic resistance in general and in tuberculosis has created an unmet need to identify novel drug targets and requires understanding of the intricate web of host-pathogen interactions during the infectious process^1^. Virulence factors of *Mycobacterium tuberculosis* (*Mtb*) are multifaceted, enabling circumvention of a dedicated immune response^2^. Herein—next to proteins—structurally complex atypical lipids are also utilized as central effectors in pathogenesis^3–7^. However, despite progress demonstrating the contribution of *Mtb* lipids to pathogenicity, relatively little is known about their mechanism of action, and more specifically, how these lipids interact with the host cell at the molecular level. The cell membrane of host immune cells is the first barrier against many pathogens and is targeted by their various evolved strategies focused towards cellular entry/exit as well as promotion of survival and infection^8^. The exhaustively studied bacterial lipid-centric mechanism in host-pathogen interaction is the functional outcome of the recognition of lipids as molecular signatures by specific host cell receptors. For example, trehalose motif of *Mtb* trehalose dimycolate (TDM) is recognized by the calcium dependent C-type lectin receptors (CLR), Mincle, triggering pro-inflammatory cytokine production^9^. Contrastingly, mannosylated moieties from mannose-capped lipoarabinomannan (LAM) are recognized by mannose and DG-SIGN receptors initiating anti-inflammatory signaling cascade that permits *Mtb* to evade immune detection^10^.

More recently the indirect mechanism of perturbing host cell function by *Mtb* lipids propose their insertion into the host membranes impairing their biophysical properties and leading to altered activities of membrane-bound effectors and modulated membrane-associated signaling^3,11^. In general, optimal membrane biophysical properties precede normal cellular functions, and thus have been subsequently manipulated by pathogens^12–14^. For example, *Mtb* lipid LAM has been shown to insert within the raft domains of the host cell membrane, modulate activities of associated effector kinases that prevent phagosome maturation and down-regulate autophagy activity^5,15,16^. *In vitro* biophysical studies on solid supported membranes furnished that incorporation of LAM in membranes modifies the morphology of the cholesterol-enriched domains and prevents vesicle fusion^17^. Surprisingly, which biophysical membrane properties are altered upon LAM incorporation still remains unknown. Similarly, TDM has been shown to insert into model and natural membranes wherein it decreases membrane fluidity and enhances permeability^18^. TDM also has been demonstrated to inhibit calcium-induced vesicle fusion, and thus implicated towards the inhibition of phagosome maturation^19^. On the other hand, insertion of glycopeptidolipids (GPL) increase membrane permeability with minor effect on fluidity^18^. Structurally unrelated *Mtb* virulent lipid, phthiocerol dimycocerosate (PDIM) has been shown to decrease membrane polarity, interpreted as changes in lipid organization in the host cell membrane leading to increased rigidity^11^. Later in an independent study, these PDIM coordinated changes were correlated to induction of host cell death via necrosis^4^, even though the molecular mechanism of this membrane reorganization, direct visualization of the same and how that is linked to PDIM-induced host cellular response remains to be elucidated.

**Figure 1:**
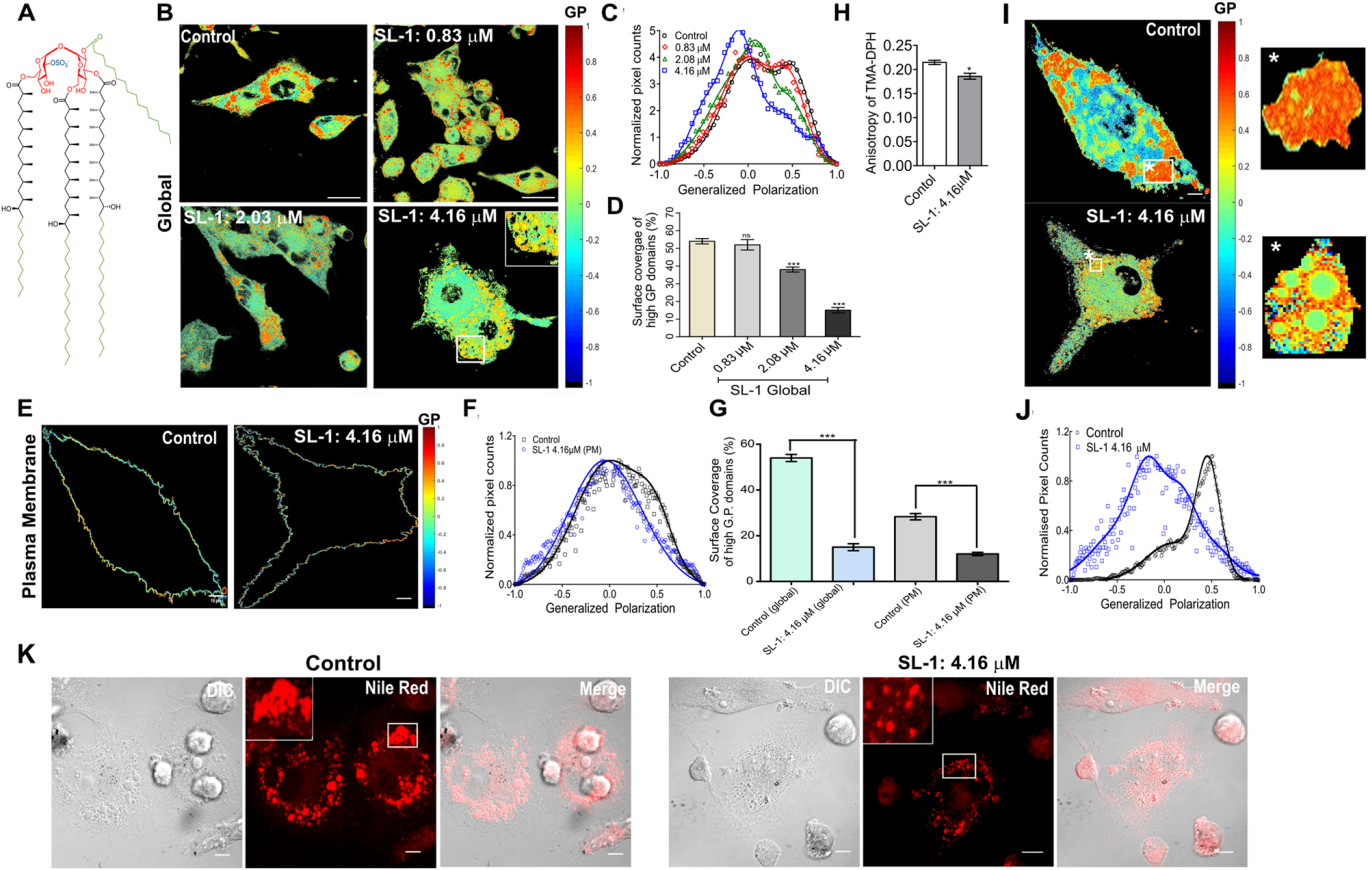
*Mtb* virulent lipids distinctly alter the host cell membrane order and fluidity. (**A**) Chemical structure of *Mtb* Sulfoglycolipid-1 (SL-1). (**B**) Pseudo-colored GP images of THP-1 macrophage control cell and cells in the presence of the indicated concentration of *Mtb* SL-1 for 4 h. (**C**) Global GP distribution from the stack of GP images (n = 90, N = 3) deconvoluted by fitting Gaussian distributions. (**D**) Surface coverage (%) of high G.P. domains (global) equated to the area under the curve of high G.P population. (**E**) Membrane segmented pseudo-colored GP pixels of control and SL-1 treated cells and (**F**) associated GP distribution fitted to Gaussian distribution. (**G**) Surface coverage (%) of high G.P. domains both globally and in plasma membrane of cells in control and in presence of 4.16 μM SL-1, equated to the area under the curve of high G.P populations. (**H**) Reduced membrane micro viscosity of SL-1 treated THP-1 cell membrane. Control and SL-1 treated THP-1 cells were labeled with TMA-DPH (4 µM) and the fluorescence anisotropy were measured. Data are mean ± SEM from three independent experiments and were compared using the two-tailed student’s *t*-test (*P < 0.01). (**I**) Pseudo-colored GP image of control and SL-1 treated cells displaying segmented objects (indicated with the white star) and the object GP image and their (**F**) associated GP distribution fitted to Gaussian distribution. Scale bar: 10 μm, 40X water objective. (**J**) Confocal fluorescent images of control and SL-1 treated cells stained with Nile red to observe lipid droplets. Scale bar 10 μm, 63X oil objective.

Collectively, the data underlines a plausible “host cell membrane insertion” mode of action of *Mtb* lipids either alone or in conjunction with other factors to facilitate infection. However, direct correlations between pathogenic lipids, the host cell membrane and the associated signaling response have not been addressed before. Moreover, the data also highlights a unique pattern of interaction of structurally diverse *Mtb* lipids with host cell membrane that leads to distinct effects including altered membrane fluidity, permeability, reorganization of lipid domains, and disruption of bilayer ordering. Given this progress, little is known about the most abundant sulfatide in the *Mtb* outer membrane, Sulfoglycolipid-1, SL-1 (Fig. 1A), a tetra-acylated trehalose-based lipid. It is uniquely expressed in pathogenic mycobacteria with its cellular abundance correlating with the strain virulence. It has been demonstrated to be released from the membrane surface of *Mtb*, thus qualifying as a plausible virulence factor to infect the host cells^20–22^. SL-1 has been proposed to modify phagosome-lysosome fusion, disrupt mitochondrial phosphorylation and activate as well as suppress cytokine levels in human leukocytes, often leading to conflicting results^20^. To directly address the role of SL-1 in tweaking host cell membrane, we characterized the biophysical, nanomechanical and cell biological properties of live THP-1 macrophage cell membranes upon interaction with SL-1. In this work, SL-1 was used at a concentration range that is physiologically relevant in context of the amount of these lipids present within the extractable pool of lipids in the outer bacterial cell membrane^23^. Our results show that the SL-1 remodels the cell membrane’s architecture, alters its nanomechanical properties and affects membrane fluidity. The effect percolates to the actin cytoskeleton underneath the membrane, and finally leads to activated autophagy signaling. This result aligns with the work by Lee *et al*., that revealed direct engagement of total *Mtb* lipids and not proteins from different bacterial strains in tempering with host autophagy^24^.

Taken together, we probed various aspects of host cell membrane-lipid interactions to generate a framework for improving our understanding of the role of *Mtb* lipids in hijacking the “host cell membrane” and manipulating the same to commandeer host membrane–associated processes. These results reinforce that an in-depth understanding of *Mtb* lipid-host membrane interactions, guided designs of structural analogs of lipids and well-described steps of membrane modulation, together, holds a promise as a lipid-based therapy. This approach also has the potential to avoid occurrence of resistant phenotype by virtue of targeting specific membrane properties.

## Results

### SL-1 mediates remodeling of the macrophage cell membrane fluidity

The lateral organization of cellular membrane is highly complex and displays varying levels of heterogeneity in lipid packing. Accumulating evidence suggests existence of liquid ordered cholesterol-enriched (l_o_) lipid raft domains alongside less organized more fluid liquid disordered (l_d_) regions in cellular membranes, potentially regulating various cellular functions such as signal transduction^25–27^. To assess the role of SL-1 in regulating cell membrane properties, we performed quantitative imaging of lipid membrane order in the cell membranes of live THP-1 macrophages using a polarity-sensitive probe, Laurdan, and two photon microscopy. Visualization of ordered (peak emission λ ~ 440 nm) and disordered fluid membrane regions (peak emission λ ~ 490 nm) was rendered by probing the degree of lipid packing, using the ratiometric measure of two emission regions known as generalized polarization, GP (Eq. 1)^28^. Collective data from model and cell membranes have shown that the fluid l_d_ regions have average GP values from ~−0.05 to 0.25 and l_o_ ordered regions vary from 0.25 to 0.55^29,30^. Pseudo-colored GP images of control THP-1 macrophages (Fig. 1B) showed, a heterogeneous membrane lipid order distribution with irregularly distributed high GP areas (colored red to orange). These areas do not represent single membrane domains, but rather cellular areas in which the fraction of ordered lipid domains is higher. Moreover, in the complex environment of cell membranes, GP values only reflect the overall membrane structure. These structures could also correspond to lipid droplets enriched in saturated lipids and cholesteryl esters and hence display high lipid order and GP values (see below)^31^. Global GP histogram of control membranes displayed a bimodal distribution centered at distinct GP values (*C1* = 0.01, and *C2* = +0.54, Table S1), indicative of co-existing fluid (46% coverage) and ordered (54% coverage) membrane regions (Fig. 1C): the latter likely to have contribution from plasma membrane due to the presence of cholesterol, compared with intracellular membranes.

Addition of SL-1 leads to a concentration-dependent perturbation of the cell membrane order. Overall membrane fluidization was evident (Figs 1B and S1A) and was also supported by the shift of the global GP distribution to lower values (Fig. 1C). The high GP domains/regions were reduced/re-distributed in a concentration-dependent manner (Fig. 1D). From the global GP distribution, the mean GP values for fluid and ordered membrane regions decreased to −0.10 (*C1*, 85% coverage) and 0.42 (*C2*, 15% coverage, Table S1), respectively. Thus, ordered regions became less abundant and less ordered, indicating membrane de-condensation, while the fluid regions became more abundant and more fluid in nature upon SL-1 interaction. Additionally, the formation of distinct domains with outer ring-like structures of relatively higher GP (than inside) was visible on cell membrane, plausibly reflecting selective lipid phase separation/de-mixing induced by SL-1 (Fig. 1B, inset). For better observation of GP changes, the difference GP distribution was plotted, which showed a SL-1 dependent decrease in GP values for ordered regions from ≈+0.1 to +0.75, and a minor increase was observed for disordered regions from ≈−0.1 to −0.8 (Fig. S1B). These regions could possibly correspond to the GP intervals reported for l_o_ (raft-like), l_d_ (non-raft), pure fluid (<−0.05) and gel (>+0.55) phases, respectively. A multimodal GP distribution, especially at the highest tested SL-1 concentration, could reflect the existence of these lipid phases in THP-1 macrophage membranes (Fig. S1C) and reflect fine-tuning of host cell membrane organization between rigid and fluid lipid phases with varying membrane order and fluidity upon bacterial lipid perturbation. Previous studies on THP-1 cells have revealed significant augmentation in the number and reorganization of rigid lipid domains under oxidative stress^32^. We thus hypothesize that the decreasing GP population corresponds mostly to disappearance of the ordered l_o_ domains. Furthermore, the temperature-dependent studies also support a significant loss of ordered membrane regions upon SL-1 interaction, displaying lower temperature sensitivity (Fig. S2). The minor increase of the low GP value population could either correspond to *de-novo* de-condensation or redistribution of fluid membrane regions.

Next, we analyzed the contribution originating from cell plasma membrane (PM) within the global GP changes, as Laurdan can also internalize and stain intracellular membranes. Laurdan GP images for THP-1 macrophages made it difficult to extract GP pixels corresponding to PM by simple visual inspection, mostly stemming from their morphology. Thus, we performed spectral imaging of cells coupled with PM segmentation to remove the cytosolic contribution of the internalized dyes^33^. GP distribution corresponding to the plasma membrane showed a unimodal distribution upon SL-1 interaction with loss of high GP regions, suggesting fluidization/reorganization of the cell plasma membrane by SL-1 (Figs 1E,F and S1D). The reduction in the ordered lipid domains corresponding to PM (2.3-fold) was lower than that observed with ordered lipid domains observed globally (3.6-fold). This indicates that SL-1 interaction does alter the membrane organization of host cell plasma membrane as well, though the effect is minor (Fig. 1G). A decreased microviscosity of the cell plasma membrane, denoted by restricted rotational mobility of the TMA-DPH membrane probe was obtained supporting the GP results (Fig. 1H). Orthogonally, using methyl-β cyclodextrin (MβCD) which causes cholesterol depletion from the cellular PM^34^ we identified that the ordered GP population discernible in the global GP distribution was most affected by this treatment, leading to a shift to lower GP values with a significant reduction of its surface coverage within 30 minutes of incubation (Fig. S3 and Table S2). This predominantly reflects the PM, where, as expected the lipid packing and order decreases upon cholesterol depletion. This indirectly suggests that the changes in the difference GP distribution (Fig. S1B) might correspond to the alterations induced by SL-1 in the host cell PM as well. Next, using the object segmentation program of the spectral imaging toolbox, we obtained a rather complex lipid environment of the circular membranous structures (Fig. 1B, inset and 1I) induced by SL-1, spanning a wide range of membrane order (Fig. 1J) compared with the control. The highly ordered red domains on the control THP-1 cells seen in global GP imaging could also correspond to intracellular lipid droplets (plausibly seen due to Laurdan internalization), as demonstrated by quite intense staining with Nile red (Fig. 1K). SL-1 treatment caused a substantial reduction in the lipid droplets (including size), possibly explaining the drastic reduction of ordered GP pixels in global GP distribution (without affecting the PM GP distribution much).

**Figure 2:**
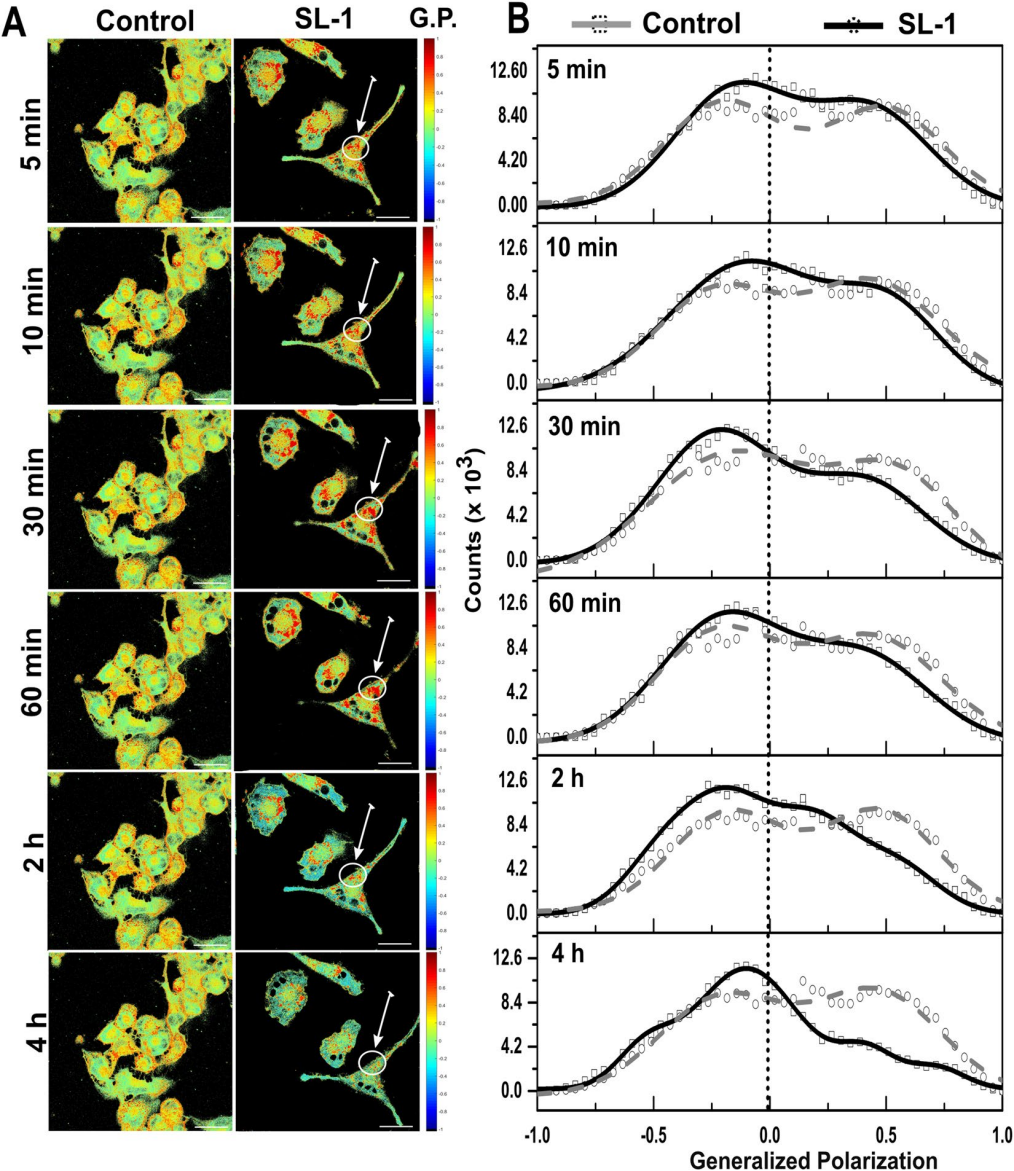
Temporal remodeling of cell membrane domains by SL-1. Pseudo-colored GP images of (**A**) THP-1 macrophage untreated cells and cells in the presence of *Mtb* SL-1 lipid at 4.16 μM. (**B**) Global distribution from the stack of GP images (n = 15, N = 3) deconvoluted by fitting Gaussian distributions. Scale bar: 10 μm, 40X water objective.

**Figure 3:**
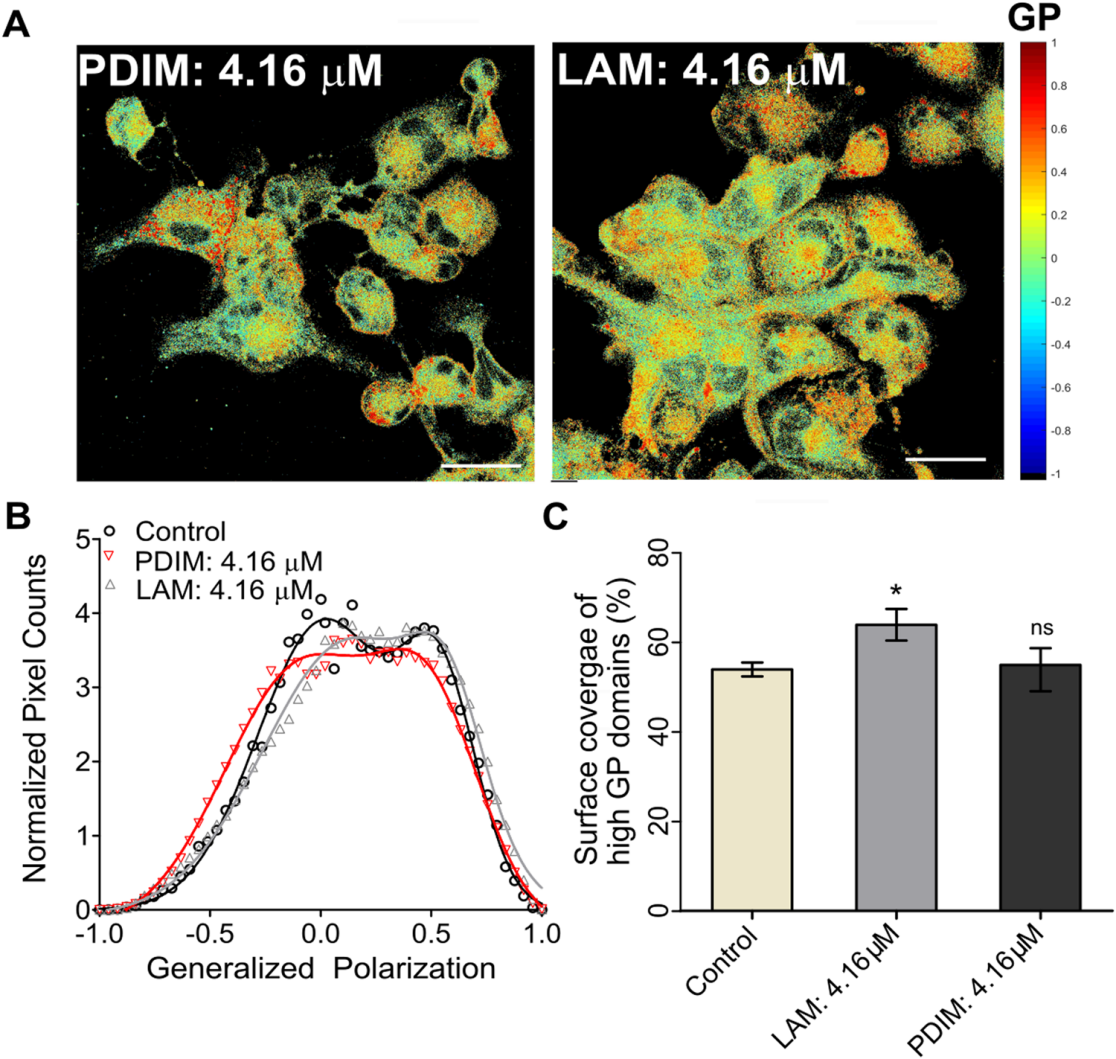
Selective cell membrane perturbation by other *Mtb* lipids. (**A**) Pseudo-colored GP images of THP-1 macrophages in presence of *Mtb* LAM and PDIM lipids. (**B**) Global distribution from the stack of GP images (n = 15, N = 3) deconvoluted by fitting Gaussian distributions. (**C**) Surface coverage (%) of high G.P. domains (global) equated to the area under the curve of high G.P. (Scale bar: 10 μm, 40X water objective.

Altogether, these data indicate that SL-1-membrane interaction leads to a substantial redistribution of lipids and plausibly proteins, causing reorganization of lipid domains and hence the host cell membranes. SL-1–membrane interaction evolved temporally with changes being observed as early as 30 min (Fig. 2 and Table S2). Initially, a transient clustering of ordered domains was seen, followed by their gradual decrease and within 2 hours, the membrane displayed gross fluidization with intermittent patterns of intermediate GP domains surrounding more ordered regions. These observations are clearly indicative of dynamic alterations in the cell membrane organization during host-pathogen crosstalk. The effect of SL-1 on the cell membrane organization was also found to be highly specific to this class of *Mtb* lipid compared with other virulent lipids such as PDIM and LAM (Figs 3A,B and S1D). PDIM did not cause any substantial change in the membrane fluidity and organization, whereas, LAM led to minor ordering of the cell membrane surface with an associated increase in the coverage of the ordered membrane region (Fig. 3C). This could also be a manifestation of varying degree of membrane partitioning of the different lipids. However, lipids with chain lengths greater than 18 carbon atoms have been shown to partition equally into phase segregated model membranes^35^. Thus, given that the major chain lengths in the investigated lipids are above C20, it might be reasonable to assume their similar incorporation efficiency into host membranes, with an only minor contribution, if any, to the observed phenotype. This underscores that the crosstalk of virulent lipids with the host cell membrane is highly selective and specific, leading to differential membrane re-organization with associated changes in membrane order and hydration, which till now remained unexplored.

### Nanomechanical cell membrane properties and membrane tether formation are perturbed by SL-1

**Figure 4:**
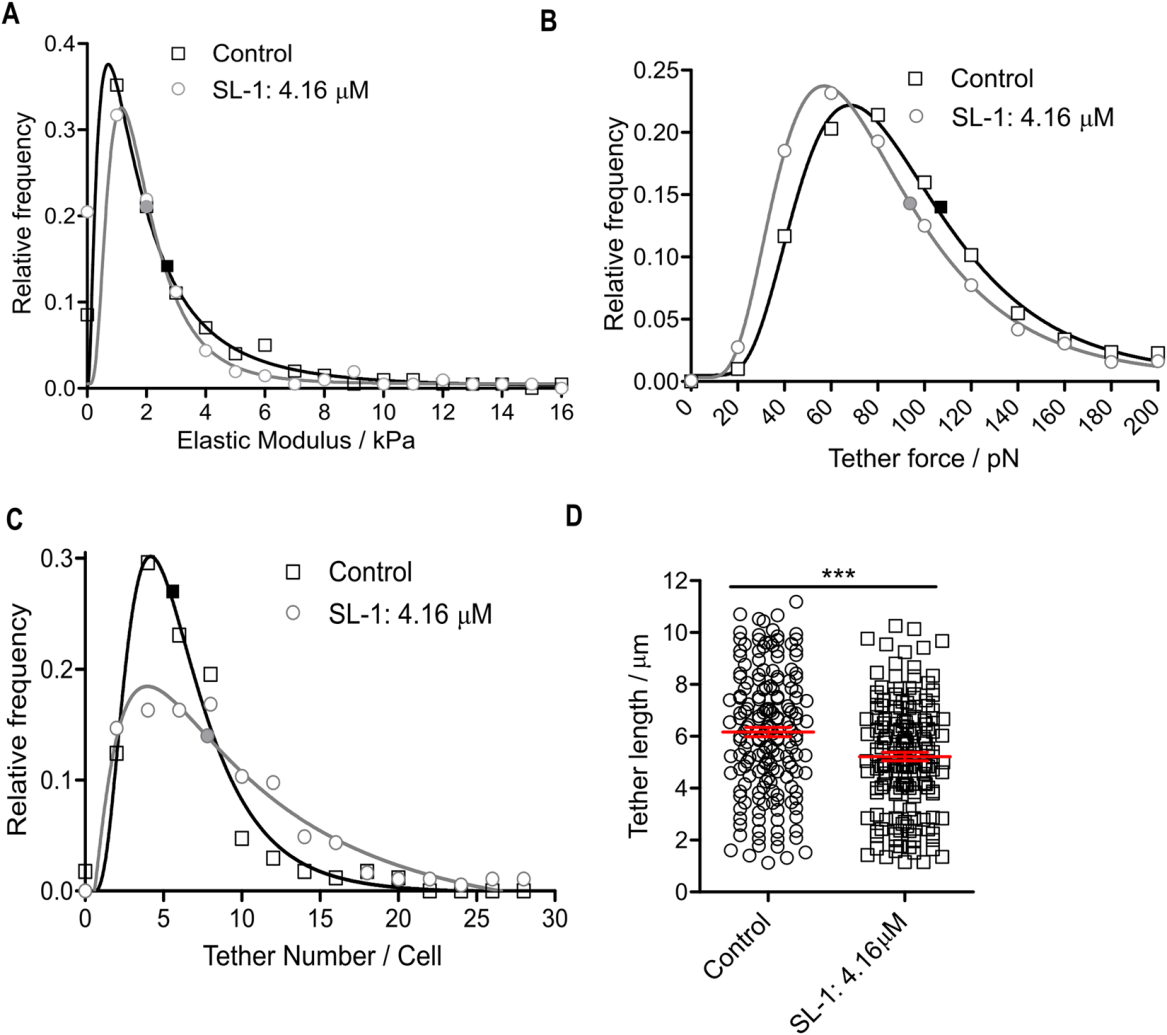
Nanomechanical membrane properties of live THP-1 cells upon lipid perturbation. (**A**) Elastic moduli distribution of THP-1 cells in the absence (n = 199 cells median: 1.74 kPa) and presence of 4.16 μM SL-1 (n = 210 cells, median: 1.44 kPa) at 2 h, fitted with a single log-normal distribution. (**B**) Relative frequency of membrane tether forces in control (n = 1182 elements, median 85.7 pN) and SL-1 treated (n = 1134 elements, median 74.4 pN) cells fitted with a log-normal distribution. (**C**) Tether number distribution of THP-1 cells in the absence (n = 169 cells, median: 5.0) and presence of 4.16 μM SL-1 (n = 184 cells, median: 7.0) fitted with a single log-normal distribution. The medians of the data in A-C were significantly different from each other (*P* < 0.05, Mann-Whitney test). (**D**) Tether length distribution of control and SL-1 treated cells (n = 175 each, mean + SEM: 6.2 ± 0.1 μm and 5.2 ± 0.2 μm, respectively) with significant mean difference (***P < 0.0001, unpaired student *t*-test).

Atomic force microscopy (AFM) is a versatile tool for mapping the nanomechanical properties of cell membranes. Membrane stiffness, especially, is reported to regulate a plethora of cellular processes, including differentiation, migration, proliferation, and is markedly altered in diseased states^12,13^. The analysis of the cortical stiffness of THP-1 macrophages revealed a significant softening upon SL-1 interaction (Fig. 4A, median elastic modulus of 1.74 kPa and 1.44 kPa for control and SL-1 treated cells, respectively; *P* < 0.05, Mann-Whitney test). These results suggest SL-1 perturbs the cell mechanical property via reduction in membrane order, increase in hydration and inducing cell cortical softening. Next, we investigated membrane tethers or nanotubes which are important regulators of cellular adhesion and communication and owe their existence to the highly dynamic PM^36^. The single-tether force distribution for SL-1 treated cells showed a significant reduction (13%) in the median tether force (74.4 pN) when compared with the control (85.7 pN) (Fig. 4B; *P* < 0.05, Mann-Whitney test). This indicates that SL-1-treated cell membrane tethers require less force to be extended, and hence their surface tension or bending rigidity/elastic modulus is smaller. The number of tethers per cell increased upon SL-1 treatment (Fig. 4C) with a median centered at 5.0 and 7.0(*P* < 0.05, Mann-Whitney test) for control and SL-1 treated cells, respectively. Furthermore, a reduction in the total extension of the last tether (i.e. tether length) was observed (Fig. 4D), 6.2 ± 0.2 μm in control and 5.2 ± 0.1 μm in SL-1 treated (mean ± SEM, *P* < 0.001, t-test). All these results point towards the cell membrane being more susceptible to tether formation governed by the effect of SL-1 on the cell mechanical properties coupled with a decreased lipid ordering as revealed by the Laurdan fluorescence studies (Fig. S1A).

### Modulation of actin cytoskeletal organization by SL-1

**Figure 5:**
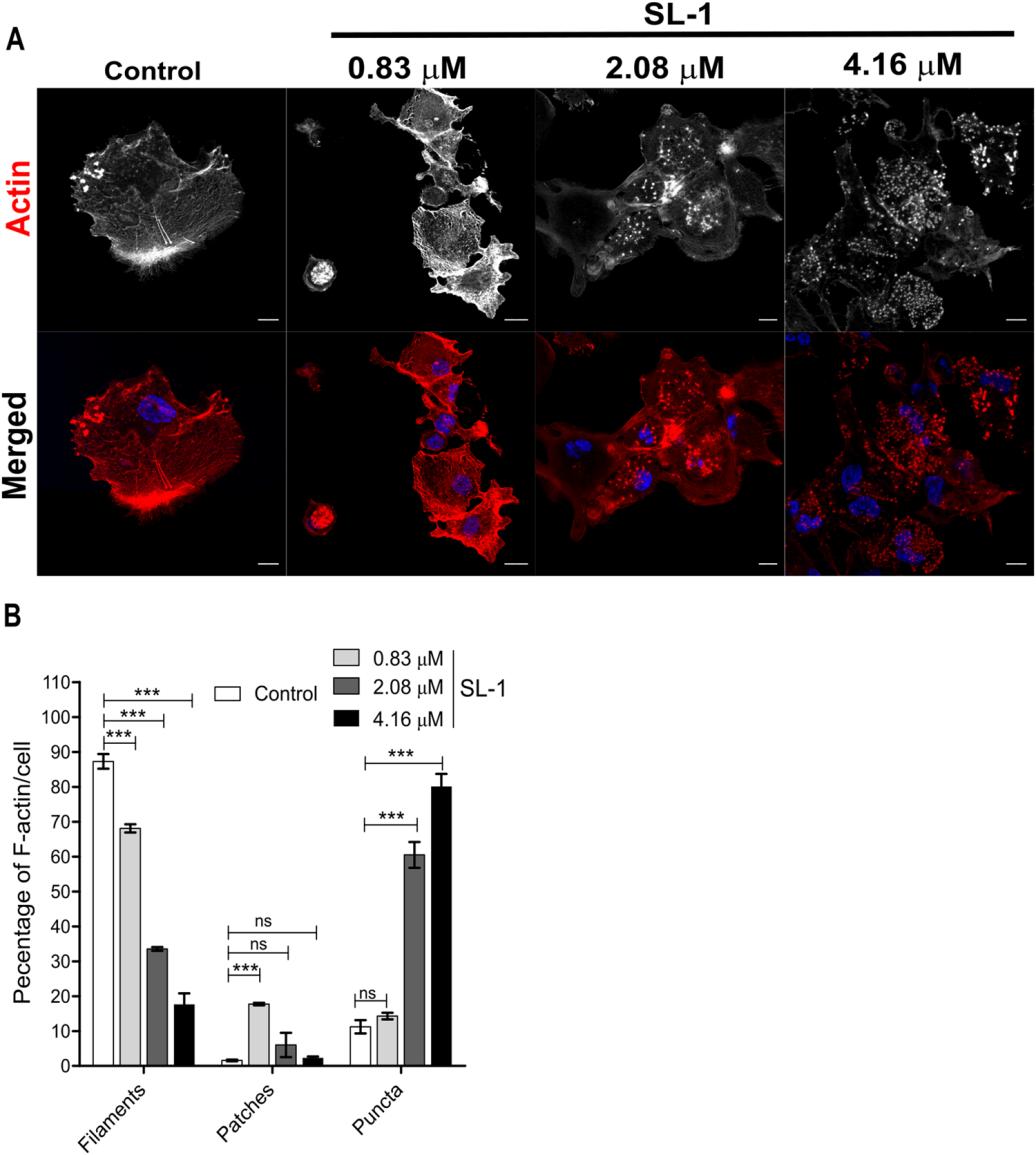
SL-1 mediates actin cytoskeleton disruption. (**A**) Representative confocal actin-labeled fluorescent images of THP-1 macrophage cells in the absence and presence of the indicated concentration of SL-1 after 4 h. Scale bar: 10 μm, 63X oil objective. (**B**) Quantitative representation of the various F-actin forms per cell from three independent biological experiments (data are mean ± SEM). ***P value < 0.001, *t*-test.

Various pathogens actively interfere with the actin cytoskeleton to trigger uptake^37^. Pathogenic mycobacteria disrupts the actin cytoskeleton by inducing its rearrangement and fragmentation. However, the exact mechanism is still unknown, and the identity of implicated moieties remain obscure^38^. Various studies have highlighted intimate crosstalk of the cell’s PM and the underlying actin filaments attuning many physiological processes^37,39^. Given these facts and our observation of dynamic reshaping of the host cell membrane, we investigated the effect of SL-1-membrane interaction on the actin cytoskeleton in THP-1 macrophages. Untreated cells displayed (Fig. 5A) at least three distinct actin morphologies^40^ – (a) thin actin filaments, 87.3 ± 2.1%, (b) F-actin cortical patches, 1.6 ± 0.3% and (c) actin punctate structures, 11.2 ± 1.9% (data are mean ± SEM). Incubation of cells with SL-1 led to a concentration-dependent increase and decrease in actin puncta and filamentous actin, respectively. The actin patches showed a non-linear response with maxima centered at the lowest SL-1 concentration tested, suggesting a transient increase in the actin patches followed by subsequent fragmentation forming punctate structures of actin (Fig. 5B). Thus, it is tempting to speculate that the pool of actin patches might act as a reservoir for the redistribution of actin into various morphologies under selective perturbation, in this case, SL-1-membrane interaction. Altogether, it can be clearly seen that the SL-1-host membrane interaction profoundly affects the actin cytoskeleton underneath the cell PM without affecting the cell viability (Fig. S4).

### SL-1 activates host-autophagy signaling

**Figure 6:**
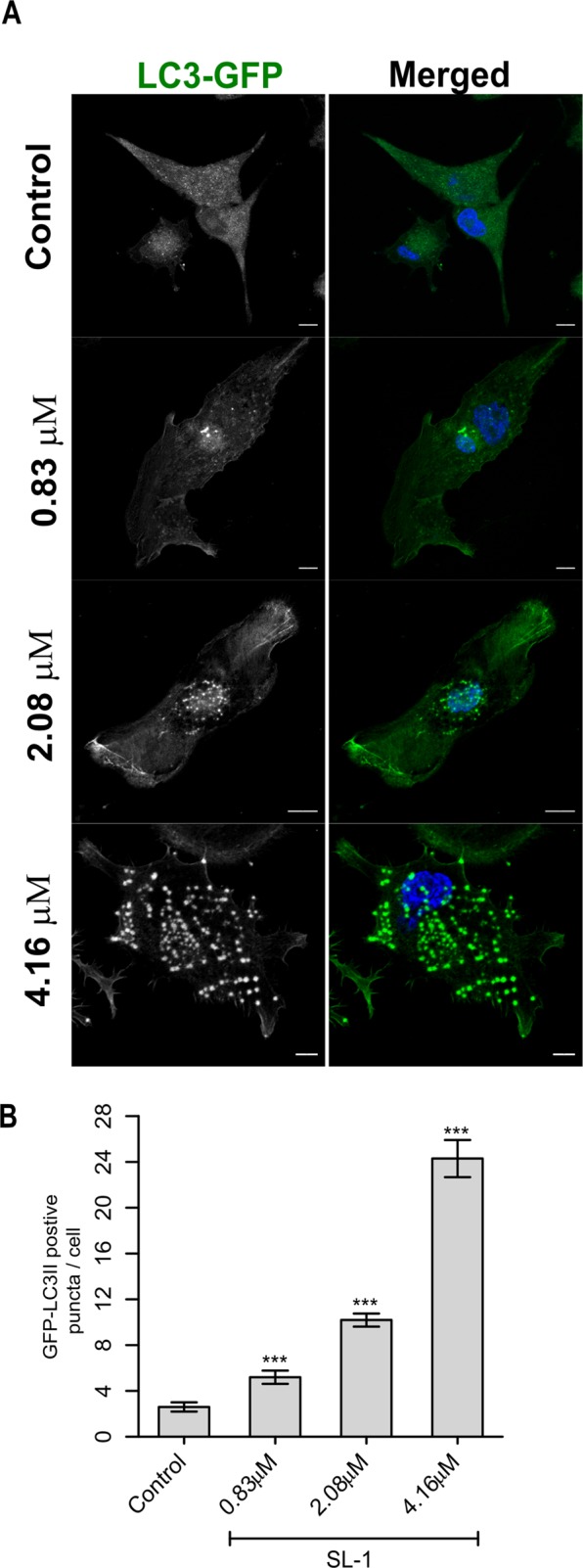
Autophagy induction in THP-1 cells by SL-1. (**A**) Representative confocal fluorescent images of THP-1 macrophages in the absence and presence of the indicated concentration of SL-1 after 4 h. Scale bar: 10 μm, 63X oil objective. (**B**) Quantitative representation of the average number of LC3-GFP positive puncta per cell from three independent biological experiments (data are mean ± SEM). ***P value < 0.001, *t*-test.

SL-1 interaction with the macrophage cell membrane led to prominent changes in the membrane biophysical properties. Hence, we characterized the differences between the membrane-associated signaling pathways—potentially dictated by cell membrane properties. The stress-response autophagy pathway—dependent upon actin and ordered lipid domains—detects intracellular pathogens, and various *Mtb* virulent strains have been documented to impair the same^24^. Intriguingly, SL-1 belongs to the family of sulfatides, the first ever reported *Mtb* component to inhibit generic phagosome-lysosome fusion and acidification, similar to that observed for a well characterized glycolipid man-LAM^40^. SL-1 was recently shown to act as a TLR-2 antagonist and perturb pattern-recognition-receptor host signaling pathways such as NF-κB and IFN^21^. These findings implicate SL-1 to perturb host cellular signaling. Thus, we sought to characterize the effect of SL-1 on the host autophagy-signaling pathway using a cell-based fluorescence assay employing LC3-green fluorescent protein (GFP). Control cells exhibited a minor abundance of LC3-GFP puncta/cell characteristic of low basal autophagy activity (Fig. 6A). SL-1 induced a significant increase (≈9.3 fold)in the LC3-GFP puncta in a concentration–dependent manner, indicative of activated autophagy-signaling (Fig. 6B; *P* < 0.0001). Thus, SL-1 communication with macrophage cell membranes potentially leads to activation of membrane-associated autophagy-signaling.

## Discussion

Cellular membranes are highly heterogeneous structures with their biological properties intimately coded by their biophysical characteristics. One such characteristic parameter essential for cellular function is membrane fluidity categorizing relative motion of molecules within the membrane. Lipid domains of distinct compositions spatially regulate membrane fluidity and influentially control lipid-protein sequestration. They generate functionally relevant platforms within the membrane and modulate the nature of downstream signaling. Alterations in membrane fluidity play a critical role in the regulation of membrane properties under physiological conditions and importantly in diseases. As such, evaluating membrane fluidity is imperative for understanding the membrane-dependent mechanisms governing various cellular processes including signaling. Evidence underpinning the indispensable involvement of specific lipid moieties in diseases is emergent^41,42^. Lipids are attractive targets exploited throughout evolution by pathogens^43,44^. Among them, *Mycobacterium tuberculosis* (*Mtb*), the causative agent for tuberculosis aptly exemplifies the use of lipids as central effectors in pathogenesis^44^, with the host cell plasma membrane as its first point of contact. However, the molecular and cellular mechanisms of their interaction with host cell membranes and the consequent modulation of membrane-associated signaling are not well understood. Thus, characterizing the molecular biophysical and cellular mechanisms underlying the crosstalk between virulent lipids and the host cell membrane along with integrated signaling is expected to deepen our understanding of the host-pathogen interactions.

In this work, we have investigated the effect of the virulent *Mtb* sulfoglycolipid, SL-1 on the physical and cell biological properties of the THP-1 macrophage cellular membranes. The work uncovers molecular mechanisms by which pathogen regulates the host cell membrane’s physical properties with associated alteration in host membrane-associated processes during infection. Using Laurdan Generalized Polarization microscopy we show that THP-1 cell membranes are heterogeneous. The distribution of GP values clearly shows the co-existence of ordered and disordered fluid regions in THP-1 cells. SL-1 from within the pool of exposed lipids on the outer *Mtb* cell envelope triggers remodeling of the THP-1 cell membrane in a time and concentration-dependent manner. The membrane reorganization was associated with increased membrane fluidity synonymous with higher hydration at the membrane interface (both globally and in PM regions) along with less restricted rotational and translational diffusion. SL-1 mediated membrane reorganization led to selective demixing of lipids and decreased the ordered regions of the cell membrane. These provide functional support to previous findings that show SL-1 to impede the innate immune response by inhibiting TLR2 and TLR1/6 heterodimers^21^, which reside within the raft-like lipid domains in the PM^45^. Surprisingly, though a dipalmitoylated SL-1 is sufficient to inhibit TLR signaling^21^, the functional role of longer and branched fatty acyl chains of SL-1 remains elusive. We hypothesize that these fatty acid chains fine-tune the host cell membrane properties, as the structure of these chains critically guide the partitioning location and kinetics of their membrane insertion. This would eventually modulate various cellular responses such as decreased TLR activity, upregulated reactive oxygen species production, and altered protein phosphorylation patterns. In this respect, investigations into the fine structure of acyl chain conformation and dynamics and its biophysical properties would help understand the SL-1 host cell membrane interaction in a more detailed manner. Finally, SL-1 reduced the highly ordered lipid droplet accumulation within the cell.

We found that the decoded pattern of membrane reorganization is selective to SL-1, as other *Mtb* virulent lipids failed to recapitulate similar effects. This is in well conformity with previous studies showing strain-specific *Mtb* lipids linked to variable cellular responses, e.g., man-LAM reduces phagosome-lysosome fusion and apoptosis, and reorganizes membrane domains to disrupt of membrane-associated signaling^15,46^. By contrast, LAM counteracts some of the effects of man-LAM, and PILAM, and activates host apoptotic response instead. Taken together, these reports highlight that the structurally diverse *Mtb* lipids act as highly selective modulators of membrane properties and signaling pathways in host cells. Next, our AFM investigations demonstrated a more compliant cell membrane of SL-1 treated cells. Membrane tethers akin to nanotubes are involved in various cellular functions, especially in immunoregulation^36^. SL-1 led to a significant decrease in the tether force, implying either alterations in bending stiffness, in-plane membrane tension or membrane-cytoskeleton interactions. As the number of tethers per cell increased upon SL-1 treatment, it entails a reduced resistance of the membrane to bend. Furthermore, generation of membrane tethers involves induction of highly positive and negative curved membrane phases around the circumference and at the base, respectively. The structural aspect of SL-1, i.e., the likely inverted cone-shape of the molecule, may foster formation of highly curved regions at the site of cell membrane insertion, thus leading to more frequent membrane nanotube generation. Such SL-1induced *de novo* curved membrane regions are expected to have unique lipid order and hydration^47^ and hence may account for the distinct global GP distribution patterns. It is well known that lipid raft domains are involved in connecting actin to the cell membrane^39^. Thus we investigated the correlation between the decreasing l_o_ regions in SL-1 treated cell membrane and the actin rearrangement. We observed a marked disorganization of the actin cytoskeleton underneath the PM by SL-1 without any effect on the cell viability. The most salient feature were the disappearance of filamentous actin, and appearance of actin punctate structures in accordance with previous studies using intact bacteria^38^. Head-to-head comparisons with previous studies on PM-cytoskeleton will distinguish the interactions regulated by pathogen invasion and lead to new mechanism grounded rationale for therapeutic targeting.

Finally, the aforementioned changes in the biophysical properties and morphology of the PM and actin cytoskeleton, respectively, led to significant alterations in a central membrane-associated process – autophagy. Autophagy endows cells with the ability to eliminate toxic cellular material and plays decisive roles in innate immunity^48^. As a result, pathogenic organisms including *Mtb* have evolved precise mechanisms to tweak host autophagy^24^. We found that the autophagy activity was considerably increased after SL-1 treatment. In light of our findings, one could hypothesize that TLR2/6 antagonist SL-1 triggers host cell autophagy to generate a protective niche preventing their detection and enhanced survival. A recent report showed that a decreased plasma membrane tension (similar to that induced by SL-1) triggers PI(4,5)P2 lipid phase separation driving the inactivation of TORC2 and hence, possibly activate autophagy^49^. We postulate that lipid phase separation guided by virulent lipid–membrane interaction modulates the host plasma membrane dynamics and organization and represents an under studied mechanism of membrane-associated TOR signaling in pathogenic infections. PI(4,5)P2 lipid links PM to actin filaments, co-localizes with lipid raft markers and is strongly implicated in host-pathogen interactions; it’s involvement in SL-1–mediated effect remains unexplored^44^.

To conclude, our data highlights lipid molecules as highly efficient effectors of pathogenesis―next to proteins. The data indicates that virulent SL-1 lipid present in the extractable lipid pool on the surface of pathogenic mycobacteria is intimately involved in host cell membrane interactions and subsequently affects membrane-associated downstream cellular functions. The proposed role of SL-1 is multifaceted and our findings establish a novel and prominent role for SL-1 in modulating specific biophysical properties of the host cell membrane such as fluidity, hydration and lipid domain re-organization, and eventually activating membrane-associated autophagy signaling. Notwithstanding, SL-1 possesses a high potential for developing anti-tuberculosis therapeutic approaches and demands a deepened molecular understanding of its mode of action coupled with identification of host mediators as potential targets.

## Methods and Materials

### Laurdan generalized polarization imaging

THP-1 cells were seeded in glass bottom dishes and differentiated using 20 nM PMA for 72 h. Cells were treated with SL-1 at the indicated concentrations and time at 37 °C and 5% CO_2_ followed by washing with phosphate buffer saline (PBS) and addition of 5 μM Laurdan for 45 min. Prior to imaging, cells were washed and imaged on a laser scanning confocal microscope (Carl Zeiss, Germany) with excitation at 780 nm with a multi-photon laser (Titanium sapphire, Coherent Radiation, CA).Two simultaneous emission images were recorded in the range of 400–460 nm and 470–530 nm. Calibration images (to calculate G factor) were measured using 5 μM Laurdan solution in DMSO. Pseudo-coloured GP images were constructed using a custom-made Matlab program described as following:

The G factor was calculated using Eq. (1), in the equation; the variables are in italics,

1 $$G=\frac{G{P}_{ref}+G{P}_{ref}G{P}_{mes}-G{P}_{mes}-1}{G{P}_{mes}+G{P}_{ref}G{P}_{mes}-G{P}_{ref}-1}$$

where, GP_ref_ is the reported reference GP value (0.207) of the Laurdan dye, and GP_mes_ is the GP value of the dye in pure DMSO measured with the experimental set-up used for experiments with cells. Two channel images, which were used to construct pseudo coloured GP images using a custom made Matlab program described below and Eq. (2),

2 $$GP=\frac{{I}_{400-460}-G{I}_{470-530}}{{I}_{400-460}+G{I}_{470-530}}$$

GP values are given in a range of −1.0 (high fluidity) to +1.0 (lowest fluidity, ordered phase) using a custom colour palette. The stacks of confocal images were converted to binary images (with background 0 and intensities above background set to 1), threshold were set and then multiplied with the respective GP images. All GP images were corrected using the G-factor as mentioned above. Laurdan pixeled counts were obtained from the Matlab based-macro and GP distributions were obtained from the histogram of the GP images, which were fitted to Gaussian functions using Origin Software (Origin Pro.9.1).

### Immunofluorescence confocal microscopy

After treatment of differentiated THP-1 cells with SL-1, cells were fixed with 4% paraformaldehyde for 10 min at room temperature (RT) and rinsed with PBS. Then, cells were permeabilized followed by blocking in 1% BSA for 1 h, rinsed with PBS/PBST. TRITC-Phalloidin (Invitrogen) was used to stain F-actin (2 μg/mL) for 30 min in dark, rinsed thoroughly with PBS and DAPI (Invitrogen, 12 μg/mL) was added. Images were taken on Laser scanning confocal microscope (Carl Zeiss) using a 63X/1.4NA objective. All post-acquisition image analysis for quantitating F-actin structures was done using ImageJ. For autophagy, briefly, differentiated cells were transiently transfected with p-GFP-LC3 plasmid using lipofectamine-3000 for 24 h followed by treatment with indicated concentrations of SL-1 for 4 h. GFP-LC3 (puncta) were quantified using ImageJ. Statistical analysis was performed using two-tailed Student’s t-test in GraphPad Prism 5.0, unless indicated otherwise. Significant differences: *p < 0.01; **p < 0.005; ***p < 0.0001. For complete cell culture conditions, transfection and lipid suspension preparation please see SI.

### Fluorescence spectroscopy

Laurdan (6-dodecanoyl-2-dimethylaminonaphthalene) and TMA-DPH (1-(4-trimethylammoniumphenyl)-6-Phenyl-1, 3, 5 p-toluene sulfonate) hexatriene were purchased from Sigma-Aldrich and Cayman Chemicals, respectively. Laurdan and TMA-DPH were dissolved in DMSO. 10^5^ cells were first treated with SL-1 for 2 hours and then trypsinized and suspended in PBS buffer and labeled with Laurdan (5 µM) or TMA-DPH (4 µM) at RT for 20 min, followed by washing (5000 rpm, 5 minutes) with PBS. The fluorescence spectroscopic measurements were performed on a Varian Eclipse fluorescence spectrophotometer with a temperature controller having an accuracy of ±0.1 °C. The temperature of the cuvette holder was adjusted to 37 °C by a circulating water bath. Laurdan was excited at 350 nm and TMA-DPH was excited at 352 nm.

For fluorescence anisotropy measurements, intensities at 440 nm and 430 nm for Laurdan and TMA-DPH, respectively, were measured with excitation and emission polarizer parallel to each other (both at 0°, I_0_) and polarizer perpendicular (excitation: 0°, emission: 90°, I_90_). The anisotropy was calculated according to Eq. (3),

3 $$r=\frac{{I}_{II}-(G\,\ast \,{I}_{\perp })}{{I}_{II}+(2G\,\ast \,{I}_{\perp })}$$

where G is the correction factor obtained from the ratio of the emission intensity at 0° and 90° with the excitation polarizer oriented at 90°.

The shift in the spectrum of Laurdan was quantified using the Generalized Polarization (GP) function defined by Eq. (4).

4 $$GP=\frac{{I}_{435}-{I}_{500}}{{I}_{435}+{I}_{500}}$$

where, I_435_ and I_500_ are the emission intensities at 435 nm and 500 nm, respectively.

### Atomic force microscopy

Force spectroscopy was performed in contact mode with MFP-3D atomic force microscope (Asylum Research) and silicon nitride cantilevers (Oxford Instruments) having nominal resonance frequency and spring constant 22 kHz and 0.16 N/m, respectively. The cantilever was calibrated using the thermal noise method (spring constant of 0.10–0.19 N/m). The applied load was 60 nN and the cantilever velocity was fixed at 2 µm/s. THP-1 cells were incubated with SL-1 suspension (4.16 µM) for 2 h followed by recording of the force curves. Elastic modulus was estimated using the Hertz model, Igor software (Asylum research). For analysis of membrane tethers (tether force, tether number and lengths), a reported custom-made Matlab program was used^9^. (for details, see the SI).

### Statistics and reproducibility

The sample sizes and statistical tests were selected based on previous studies with similar methodologies. All experiments were repeated at least three times (AFM experiments were conducted twice due to limited SL-1 amount), giving similar results. The results of independent experiments are presented as mean or median values; error bars represent SEM. Statistical significance was tested using either using unpaired two-tailed *t*-test for all data showing normal distribution. AFM results demonstrated a non-normal distribution (Fig. 4A–C) and thus were statistically compared using non-parametric unpaired Mann-Whitney test to compare medians. All data with a *P* value < 0.05 were considered significant.

## Supplementary information

Supplementary Information

## Data Availability

Source data for Figs 1–3 is provided in Supplementary Tables 1 and 2. All other data that support the findings of this study are available from the corresponding author upon request.
