# Supplementary material for: Dynamic Remodeling of the Host Cell Membrane by Virulent Mycobacterial Sulfoglycolipid-1
Source: Sci Rep. 2019 Sep 6;9:12844. doi: 10.1038/s41598-019-49343-2 (PMC6731295; doi:10.1038/s41598-019-49343-2)
Supplement: Supplementary file 1 — Supplementary Information [file 41598_2019_49343_MOESM1_ESM.pdf]

Supplementary Information

Dynamic Remodeling of the Host Cell Membrane by Virulent Mycobacterial Sulfoglycolipid-1

Manjari Mishra, PranavAdhyapak, Ruchika Dadhich, and Shobhna Kapoor*

Department of Chemistry, Indian Institute of Technology Bombay, Powai, India.

* Correspondence: Prof. Dr. Shobhna Kapoor
Membrane Biophysics and Chemical Biology Lab,
Department of Chemistry,
Indian Institute of Technology Bombay, Powai – 400076, India
Tel: +91 (22) 25767161
E-mail: shobhnakapoor@chem.iitb.ac.in

This supporting information appendix contains SI materials and methods, Tables S1-S2, Figures S1 to S4 and references.

Materials and Methods:

Cell lines, *Mycobacterial* lipids, plasmid and reagents

Fetal bovine serum (FBS) was procured from HyClone (USA), Roswell Park Memorial Institute medium (RPMI-1640), 1X Dulbecco's phosphate buffer saline (D-PBS), sodium bicarbonate, 20X Antibiotic-antimycotic solution, amphotericin-B, streptomycin, pencillin G, 0.25% trypsin-EDTA, cell freezing solution, methyl-cyclodextrin, Laurdan, paraformaldehyde, serum free opti-MEM, PMA (phorbol 12-myristate-13-acetate) and solubilizing agent were purchased from Sigma-Aldrich. Phallodin-TRITC and Lipofectamine-3000 were from Invitrogen. TritonX-100 was from MP Biomedicals, and plasmid puro-GFP-LC3 (Addegene: 22405) was a kind gift from Prof. Anirban Banerjee (BSBE department, IIT Bombay). THP-1 cells were a kind gift from Prof. Sarika Mehra (Chemical Engineering, IIT Bombay). Rapamycin was from Cayman Chemicals, USA. THP-1 cells were maintained in RPMI-1640 medium supplemented with 10 % heat-inactivated FBS and penicillin (100 U/ml), streptomycin (100 mg/ml), and gentamycin (20 µg/ml) at 37 °C in humidified air containing 5 % CO_2. THP-1 monocyte was cultured at a lower density (2.5 × 10^5/ml) with media being refreshed every three days for one week (as per recommended on ATCC site). For the experiments, THP-1 cells were using 20 nM PMA for 72 h. Non-adherent cells were removed by washing with RPMI without FBS. The cells were then further incubated in complete RPMI media containing no PMA for 24 h before using it

for any experiments. All experiments were performed within the cell passages [18-20]. Cells were routinely tested for Mycoplasma and found free of contamination. Purified Sulfolipid-1 (NR-14845), Phthiocerol Dimycocerosate (NR-20328) and Lipoarabinomannan (NR-14848) from *Mycobacterium tuberculosis*, Strain H37Rv were obtained through obtained through BEI Resources, NIAID, NIH.

Preparation of *Mycobacterial* lipid suspensions

Lipid suspensions were prepared by gentle hydration and freeze thaw method reported previously.(1) Lipids were dissolved in a chloroform:methanol (2:1, v/v)mixture at a final concentration of (0.5 mg/ml) and stored at -80° until further use. Lipid solutions were dried overnight to form thin films with the indicated concentrations and remove the traces of organic solvent. Next day, the dried lipid films were hydrated with media and then subjected to bath sonication for 10 min. They were vigorously shaken with a vortexer for 1 min to produce lipid suspensions, which were frozen at liquid nitrogen temperature for 1 min and thawed in a water bath at 65 ^{o}C for 5min. After 5 cycles of freeze and thaw the lipid suspensions were used for the cell treatment.

Transient transfection, autophagy experiments and MTT cell viability assays

Differentiated THP-1 macrophages seeded on glass cover slips were transfected with puro-GFP-LC3 using Lipofectamine-3000 according to the manufacturer's instructions for 24 h. Expression and function were confirmed by the formation of LC3-GFP puncta in response to positive control rapamycin (20 nM), an mTOR inhibitor. Transfected cells were treated with the indicated concentrations of SL-1 for 4 h at 37°C and 5% CO_2. Fluorescence imaging was performed using a LSM confocal microscope and GFP-LC3 (punctate) were quantified using ITCN plugin in ImageJ software. (2) Quantification was done by calculating the number of cells showing the punctate pattern of GFP-LC3 and dividing by the total number of GFP-positive cells. A minimum of 70 cells from randomly selected fields was scored per condition per experiment. Cell viability was accessed through the MTT assay. After 4h of SL-1 treatment, the medium from each well was removed and washed 2-3 times with 1x PBS. 100 μL of fresh experimental medium and 10 μL MTT (5 mg/mL) was added to each well. The plate was incubated for 3 h at 37°C subsequently, 200 μL of DMSO was added to each well, swirled gently and was left for 20 min in dark at RT. Cytotoxicity was quantified by measuring absorbance at 570 nm and 630nm (reference wavelength) using an Elisa plate reader (Cole Parmer, Germany). Data was normalized to control cells set as 100 %.

Atomic Force Microscopy

Force spectroscopy experiments were performed in the contact mode with MFP-3D atomic force microscope (Asylum Research, Santa Barbara, CA, USA) using silicon nitride cantilevers (Oxford Instruments) with nominal resonance frequency and spring constant 22 kHz and 0.16 N/m respectively. The cantilever was calibrated using the thermal noise method to obtain a spring constant of 0.10-0.19 N/m. The applied load was 60 nN and the cantilever velocity was fixed at 2 μm/s. THP-1 cells were incubated with SL-1 (4.16 μM) for 2 hours. Force curves were recorded only at the peripheral region of the cells. On each cell, at least 2

force curves were recorded. In each set of independent experiment, at least 50 cells were used for force spectroscopy. For determination of the elastic modulus using the Hertz model, Igor software (Asylum research) was used after providing necessary inputs for tip geometry and Poisson's ratio of the sample. For analysis of membrane tethers (tether force, tether number and lengths), a modified version of a reported custom-made Matlab program was used. Tethers are observed as well-defined plateaus of constant force in a force-distance curve, where the stepwise force change corresponds to the extension and rupture of individual tethers. We have considered membrane rupture events followed by a force plateau with a distance of more than 2 µm as tethers in the analysis.

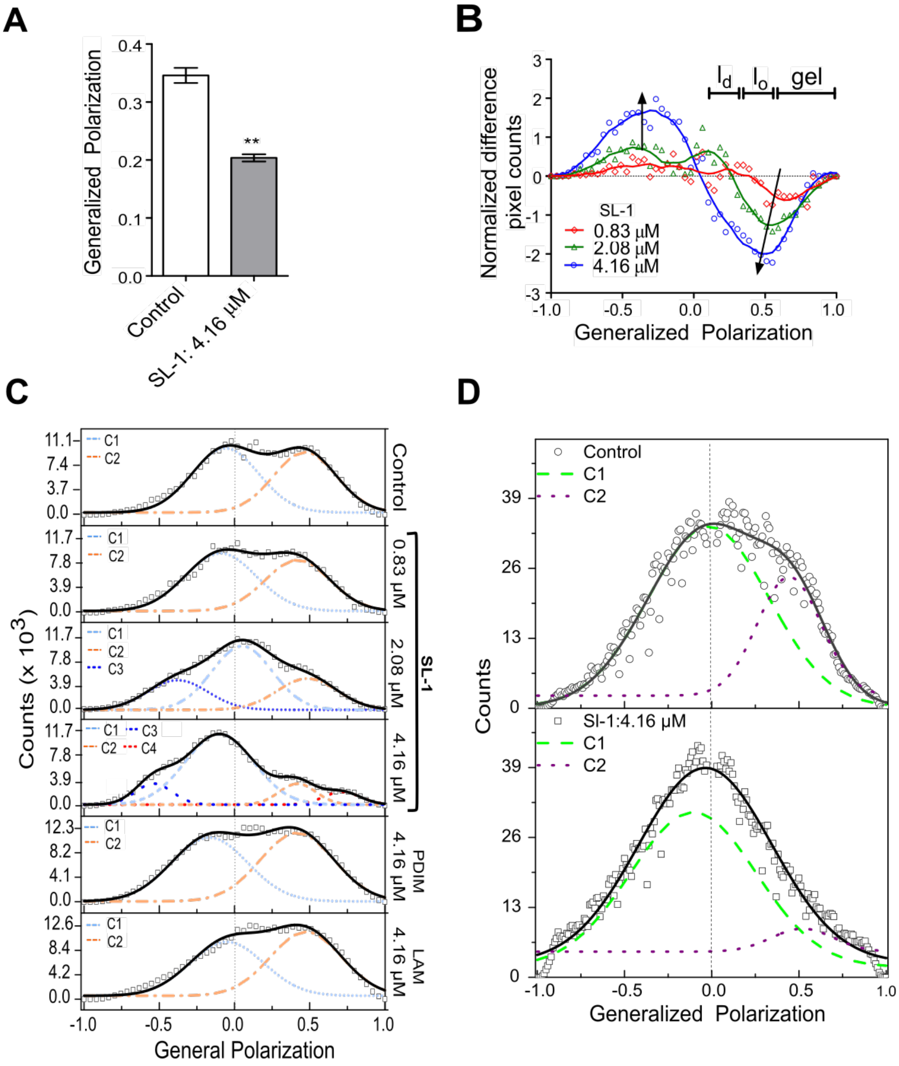

Figure S1:Membrane-modulating effect of the virulent *Mtb* Sulfoglycolipid-1; SL-1.
(A) Control and SL-1 treated THP-1 cells were labeled Laurdan (5 µM) and the fluorescence general polarization index was measured. Data are mean ± SEM from three independent experiments (***P*< 0.05)**. (B)** The global GP distribution values of control untreated cells were subtracted from the corresponding GP distribution values of SL-1 treated cells after 4 h of incubation at the indicated concentrations to obtain the difference GP distribution curves, which were further smoothened using a Savitsky-Golay algorithm. (C) Multimodal raw GP distribution of SL-1 treated cells (black solid line) at the indicated concentration, which was

fitted using multiple Gaussian distributions (dashed lines). (D) Distribution from the stack of GP (global) images (n = 90, N= 3) deconvoluted by fitting Gaussian distributions. (E) Distribution from the stack of GP (plasma membrane segmented) images (n = 30, N= 3) deconvoluted by fitting Gaussian distributions.

Table S1: **Quantitative parameters from the Laurdan GP imaging of THP-1 macrophages.**

Gaussian distributions of deconvoluted stacks of GP images of live THP-1 macrophage cells in the absence and presence of the indicated concentration of *Mtb* SL-1. Data shows peak centroid (*C1-C4*), width (*W2*) and surface coverage (area under the peak) as percentage of all Laurdan-stained pixels for respective populations. SEM is indicated in parenthesis.

	C1	*W1*	*C2*	*W2*	*Surface coverage C2 (%)*	*C3*	*W3*	*C4*	*W4*
Untreated	0.01 (0.001)	0.71 (0.03)	0.54 (0.01)	0.39 (0.02)	54.0 (2.67)	NA	NA	NA	NA
Conc.	SL-1 treated Cells								
0.83μM	-0.01 (0.02)	0.72 (0.04)	0.49 (0.01)	0.41 (0.03)	52.0 (5.09)	NA	NA	NA	NA
2.85 μM	0.05 (0.008)	0.47 (0.01)	0.48 (0.01)	0.47 (0.01)	38.0 (3.69)	-0.37 (0.01)	0.47 (0.01)	NA	NA
4.16 μM	-0.10 (0.002)	0.53 (0.02)	0.42 (0.01)	0.28 (0.03)	15.0 (2.68)	-0.53 (0.009)	0.29 (0.001)	0.71 (0.02)	0.29 (0.001)
Other Lipids									
PDIM (4.16μM)	-0.19 (0.02)	0.57 (0.006)	0.41 (0.04)	0.55 (0.02)	55.0 (4.8)	NA	NA	NA	NA
LAM (4.16μM)	-0.23 (0.12)	0.55 (0.03)	0.36 (0.06)	0.685 (0.01)	64.0 (3.52)	NA	NA	NA	NA

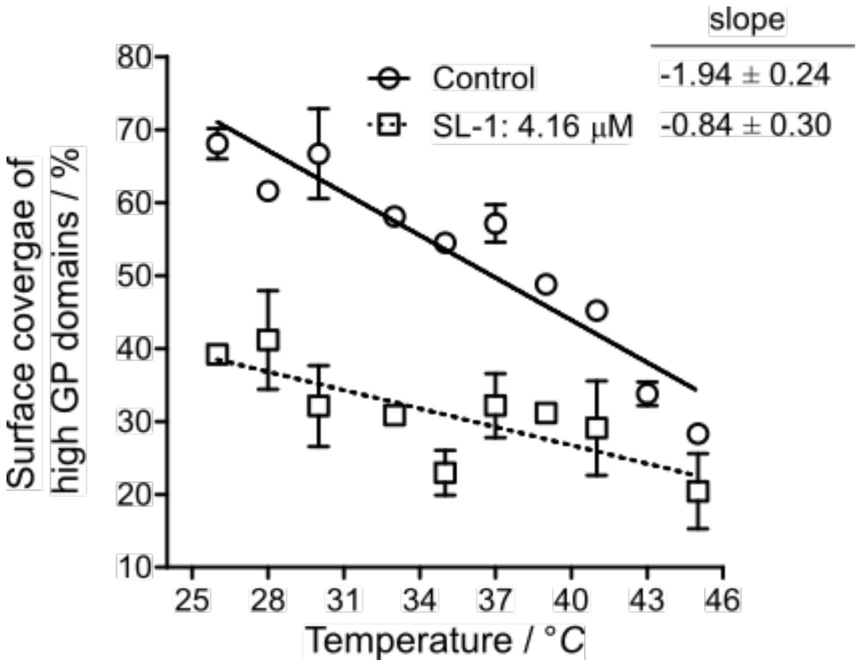

Figure S2. Differential temperature sensitivities of the lipid domains in control and Sl-1 treated live THP-1 macrophage cells.

Temperature-dependency of the surface coverage of high GP domain population (Table S1) as percent of all Laurdan-stained pixels in the presence and absence of the indicated concentration of SL-1 incubated for 4 h. Data represented are mean ± SEM from two independent experiments (n = 15, N =2). An approximately two-fold lower slope in SL-1 treated cells indicates a lower temperature sensitive behavior of ordered lipid domains on the macrophage cell surface.

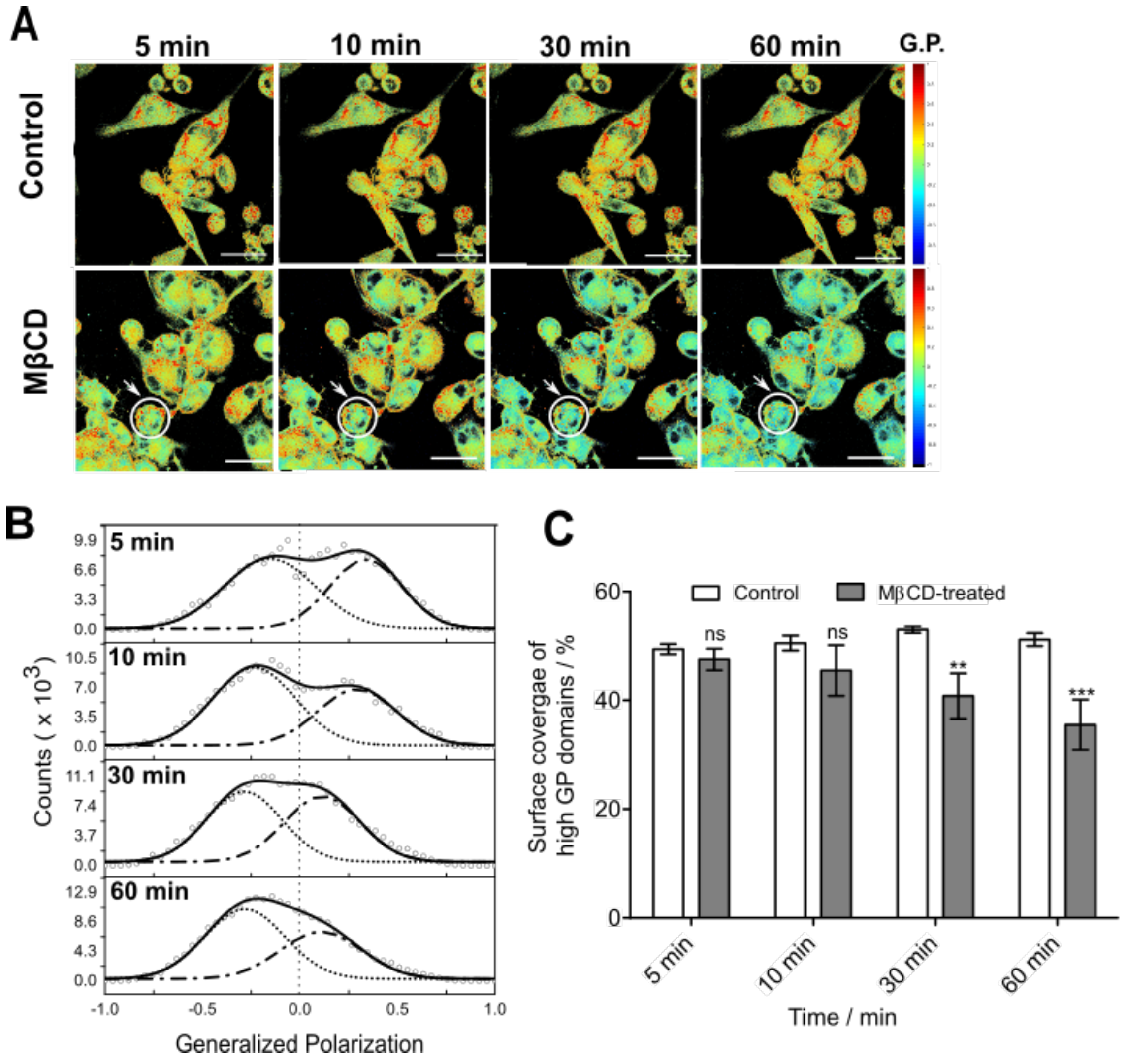

Figure S3: **Cholesterol depletion alters the high GP domains in live THP-1 cells.**

(A) GP images of THP-1 macrophage cells treated with 5 mM MβCD at 37 ^oC for the indicated times. (B) Global GP distribution (solid lines) from the stack of GP images of the indicated conditions (n = 15, N= 3) deconvoluted by fitting Gaussian distributions to the experimental data set (dashed lines). (C) Surface coverage of the high GP domains equated to the area under the curve of the high GP (*C2* centroid at GP = 0.42) population. Scale bar: 10 μm, 40 X water objective. Different conditions were compared with two-tailed student's t-test. ns: not significant, $**P< 0.005$; $***P < 0.0001$.

Table S2: **Quantitative parameters from the Laurdan GP imaging of THP-macrophages with time**

Gaussian distributions of deconvoluted stacks of GP images of live THP-1 macrophage cells in the absence and presence of MβCD, and the indicated concentration of *Mtb* SL-1. Data shows peak centroid (*C1-C2*) and surface coverage (area under the peak) as percentage of all Laurdan-stained pixels. SEM is indicated in parenthesis.

A.

	Untreated cells			MβCD-treated		
Time (min)	*C1*	*C2*	*Surface coverage (C2%)*	*C1*	*C2*	*Surface coverage (C2%)*
5	-0.03 (0.06)	0.49 (0.05)	49.4 (0.94)	-0.15 (0.03)	0.33 (0.04)	47.5 (1.98)
10	-0.06 (0.02)	0.52 (0.02)	50.6 (1.36)	-0.25 (0.01)	0.27 (0.04)	4155 (2.70)
30	-0.05 (0.06)	0.47 (0.03)	53.0 (0.59)	-0.28 (0.02)	0.13 (0.02)	40.8 (4.16)
60	-0.05 (0.03)	0.47 (0.02)	51.2 (1.2)	-0.28 (0.03)	0.1 (0.06)	35.4 (4.58)

B.

SL-1 Treated						
	0.83 μM			2.08 μM		
Time (min)	*C1*	*C2*	*Surface Coverage (C2%)*	*C1*	*C2*	*Surface Coverage (C2%)*
5	-0.11 (0.01)	0.48 (0.01)	39.6 (7.2)	-0.12 (0.01)	0.47 (0.006)	45.8 (2.9)
10	-0.11 (0.03)	0.47 (0.03)	48.5 (0.64)	-0.12 (0.04)	0.45 (0.006)	45.6 (0.57)
30	-0.19 (0.05)	0.44 (0.02)	52.0 (5.69)	-0.13 (0.02)	0.47 (0.02)	47.2 (1.25)
60	-0.12 (0.01)	0.46 (0.01)	45.0 (0.39)	-0.14 (0.02)	0.45 (0.01)	48.96 (2.56)
120	-0.16 (0.01)	0.49 (0.008)	44.9 (1.2)	-0.18 (0.02)	0.43 (0.04)	43.01 (8.45)
240	-0.13 (0.03)	0.45 (0.03)	40.06 (4.21)	-0.19 (0.03)	0.31 (0.01)	37.2 (4.47)

C.

SL-1 Treated					
4.16 μM					
Time	*C1*	*C2*	*C3*	*C4*	*Surface Coverage (C2%)*

(min)					
5	-0.15 (0.04)	0.43 (0.004)	NA	NA	48.04 (3.39)
10	-0.17 (0.04)	0.40 (0.03)	NA	NA	44.36 (1.95)
30	-0.22 (0.03)	0.41 (0.01)	NA	NA	42.2 (10.6)
60	-0.18 (0.01)	0.43 (0.01)	NA	NA	35.9 (4.2)
120	-0.20 (0.01)	0.42 (0.008)	NA	0.55 (0.001)	31.9 (2.6)
240	-0.10 (0.03)	0.43 (0.03)	-0.53 (0.04)	0.74 (0.05)	28.2 (5.2)

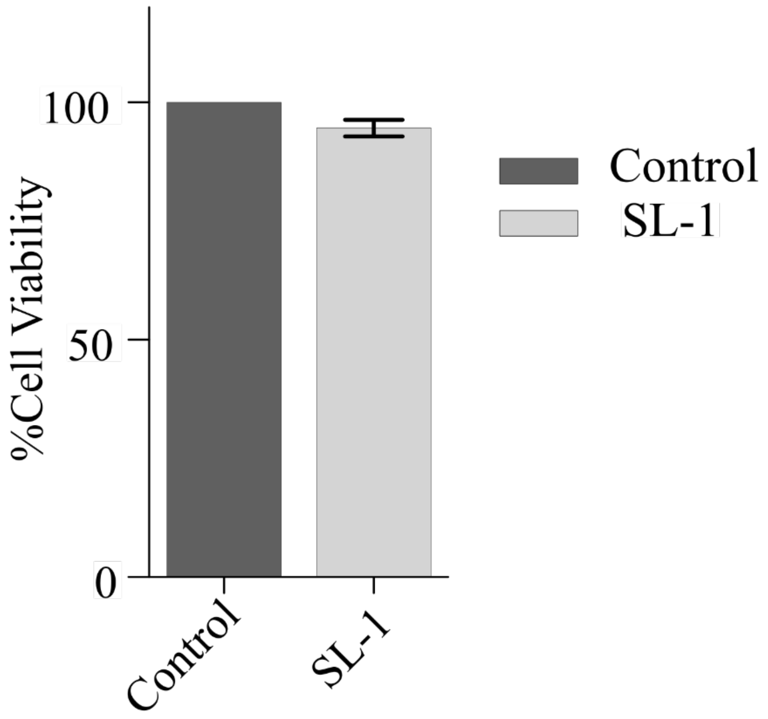

Figure S4: SL-1 does not induce alter the cell viability. Viability of SL-1 treated (10 μg/L, 4 h) and controls cells were assays using MTT reagent and depicted after normalization to control cells set as 100 %. Data presented is mean ± SEM of three independent experiments done in duplicates.

SI References

1. Kapoor, S. *et al.* Revealing conformational substates of lipidated N-Ras protein by pressure modulation. *Proc Natl Acad Sci U S A* **109**, 460-465 (2012).

2. Schneider, C.A., Rasband, W.S. & Eliceiri, K.W. NIH Image to ImageJ: 25 years of image analysis. *Nat Methods* **9**, 671-675 (2012).
